# Dr. Waugh on the Cerebro-Spinal Phenomena

**Published:** 1839-01

**Authors:** 


### Art. VI.—*The Science of the Cerebro-spinal Phenomena attempted.* By John S. Waugh, m.d.—*London*, 1838. 12mo. pp. 172.

We have, in the course of our labours, had occasion to comment on the medical literature of many foreign states, and have shown that the languages of France, Germany, Denmark, Sweden, Russia, Italy, Spain, and Portugal are alike familiar to us with our mother-tongue. We will undertake to learn those of Persia, China, New Zealand, and Tahiti, when we receive any medical works for review from those countries: but we must confess our unwillingness to devote our valuable time to the acquirement of a new language only employed by its inventor, and for the value of which we have only his own word. We cannot help thinking that the author of the volume before us is desirous of concealing for a time the grand discoveries which he professes to have set forth in it, as learned men did of old in their anagrams and riddles; and that we shall, when they are fully ripe, be favoured with an explanation of them in plain English. We beg to present to our readers the following specimen of the new language, extracted from the preface, in which we should have expected to find little but ordinary diction, or, at any rate, to be gradually introduced to the author's nomenclature.

"The cerebro-spinal influence, flowing by cerebro-dëal filaments into the sympathetic ganglions, is entirely unspecial as to the functions of these centres. The arrangement and connexion of sympa-versal and of sympa-dëal filaments in a sympathetic ganglion are altogether independent of the cerebro-spinal fasciculus, which conducts such unspecial influence to such ganglion." (p. xvii.) . . . . "The laws of transition of the unspecial perceptive oli-versal filaments, and of non-transition of the unspecial protective versal filaments, inducted by analogy from the laws of transition and non-transition of the special filaments of the same orders, furnished me with a most satisfactory explanation, not only of the curvilinear lines on each side of the median line of the corpus callosum, but also of the lyre, and the longitudinal channels on the edges of the fornix." (p. iii.)

The author tells us that, "having discovered a general law, he was able, on several occasions, to predict from it the ultimate destination of fasciculi, and much minute anatomy in the cerebro-spinal mass, of which he had previously been unaware, but the truth of which was afterwards confirmed by the descriptions of those who had no theories to support, but detailed without prejudice what they saw. The capability of such predictions is an attribute of true science." (p. ii.) Animated by these promises of a rich harvest of discovery, we were addressing ourselves seriously to the study of Dr. Waugh's enigmatical production, when we met with the following passage:

"There are many anatomical, physiological, and pathological considerations in support of the semilunar ganglion being the organ of residence of the soul of man, or where this is impressed by the perceptive influence, which I have not space here to dwell upon." (p. xx.)

On reading this, we were immediately seized with a strange feeling in the præcordial region, which, before we were enlightened by Dr. Waugh as to the residence of the soul, we should have mistaken for nausea. This feeling, whatever it may be, obliged us to lay down the book: and it has returned whenever we have opened it, so that we have, from due consideration of our own health, been compelled to abandon the task. Should any of our less sensitive readers, however, be inclined to furnish us with a brief and intelligible analysis of Dr. Waugh's views, (in which we doubt not that something worth the examination may be detected,) we will do our best to make them public.

